# Gray matter declines with age and hearing loss, but is partially maintained in tinnitus

**DOI:** 10.1038/s41598-020-78571-0

**Published:** 2020-12-11

**Authors:** Elouise A. Koops, Emile de Kleine, Pim van Dijk

**Affiliations:** 1 Department of Otorhinolaryngology/Head and Neck Surgery, University Medical Center Groningen, University of Groningen, P.O. Box 30.001, 9700 RB Groningen, The Netherlands; 2 Graduate School of Medical Sciences (Research School of Behavioural and Cognitive Neurosciences), University of Groningen, Groningen, The Netherlands; 3 Cognitive Neuroscience Center Groningen, University of Groningen, Groningen, The Netherlands

**Keywords:** Auditory system, Cognitive neuroscience, Sensory processing, Neuroscience, Anatomy, Nervous system, Brain

## Abstract

The impact of age-related hearing loss extends beyond the auditory pathway and impacts brain areas related to cognitive impairment and even dementia. The presence of tinnitus, a sensation of sound that frequently co-occurs with hearing loss, is additionally linked to cognitive decline. Interestingly, structural neuroimaging studies have reported that hearing loss may precede or modulate the onset of cognitive impairment. In this study, we aimed to disentangle the effects of age, hearing loss, and tinnitus on gray matter structure. In total, 39 participants with hearing loss and tinnitus, 21 with hearing loss but without tinnitus, and 39 controls were included in this voxel- and surface-based morphometry MRI study. Whole brain volume and surface thickness measures were compared between the groups. Age-related gray matter volume decline was observed in all groups. Several brain areas showed smaller gray matter volume and cortical surface thickness in hearing loss without tinnitus, relative to controls. This reduction was observed both within and outside of the auditory pathway. Interestingly, these reductions were not observed in participants with tinnitus, who had similar hearing loss and were of similar age. Since we have tools to improve hearing loss, hearing screening may aid in the battle against cognitive decline.

## Introduction

Hearing loss is the most prevalent acquired sensory impairment and it affects a large part of the ageing population worldwide^1^. Hearing loss not only has an impact on the quality of hearing, it is also associated with an increased likelihood of cognitive impairments and even dementia. Presently, with a globally ageing population, the prevalence of hearing loss is rising^2^ together with the interest in the impact of acquired and age-related hearing loss on the brain. In light of this, it has become clear that sensory impairments are not restricted to the peripheral sensory organs, i.e. the ear, and changes to the brain are involved too^3–6^. Correspondingly, hearing loss related brain changes appear to affect other faculties than hearing. Several studies have reported a relation between age-related hearing loss, or presbycusis, and poorer cognitive functioning and even dementia^7–13^. For instance, longitudinal data showed that hearing impairment is associated with a larger likelihood of developing cognitive impairment^7^. Furthermore, a recent study identified hearing loss as an independent modifiable risk factor for cognitive decline^10^. For a comprehensive overview, see Jafari et al., 2019^14^. It thus appears that hearing loss and cognitive decline do not only appear in the same population, hearing loss may precede or modulate the onset of cognitive impairment. Therefore, the effect of hearing loss on the brain is a potential window to understand cortical changes related to ageing.

Tinnitus is a symptom that often co-occurs with hearing loss, and is characterized by the perception of sound in the absence of an external acoustic stimulus. Since there is a strong link between hearing loss and neural plasticity and a strong association between hearing loss and tinnitus, it has been hypothesized that tinnitus is linked to neuroplasticity as well^15–20^. Furthermore, similar to hearing loss, tinnitus has been linked to an increased risk of cognitive deficits^21^. Several studies indicate that the presence of tinnitus has a negative impact on memory, executive functioning, and quality of life^22^. Interestingly, one study noted that the extent of the cognitive deficits is linked to the perceived severity of the tinnitus^21^. Thus, in addition to the strong link between tinnitus and hearing loss, it has been reported that tinnitus by itself has a negative impact on cognitive functioning. Therefore, the factor of tinnitus has to be considered when investigating cortical changes in a population with hearing loss.

To date, neuro-imaging studies identified several changes in gray-matter brain volume related to presbycusis (i.e. age-related hearing loss), either with Voxel-Based-Morphometry (VBM), Surface-Based-Morphometry (SBM) or other types of automated morphometry. The reported affected areas extend beyond the auditory pathway and correspond to areas related to cognitive impairment and dementia. In both unilateral and bilateral hearing loss, the decrease in brain volume in the auditory cortex, anterior cingulate, and prefrontal cortex correlated with the loss in hearing sensitivity^23–27^. Furthermore, VBM studies that investigated cognitive decline and presbycusis reported accelerated cortical volume decline in the presence of presbycusis^25,26,28,29^. In line with this, the results of SBM studies indicated a relationship between cognitive impairment and presbycusis. These surface-based studies identified gray matter atrophy in the primary auditory cortex, the cingulate cortex, the precuneus, the insula, and the parietal cortex in the presence of presbycusis^29,30^. Surprisingly, one study reported an association between hearing loss and an increase in the gray matter of the right angular gyrus^31^. Nevertheless, pervasive decreases in gray matter volume and surface thickness, which are generally associated with cognitive decline, may additionally be linked to age-related hearing loss.

Furthermore, recent neuroimaging studies have tried to identify the central correlate of tinnitus. These studies identified changes in gray matter volume in auditory, prefrontal, and limbic areas that were associated with the presence of tinnitus or its characteristics. More specifically, reductions in gray matter were reported for the prefrontal cortex^32–37^, the subcallosal area^35,38^, precuneus^32^, supra marginal gyrus^33^, orbitofrontal cortices^39^, cingulate^36^, insular cortex^36^, and the pre- and postcentral gyrus^36^. However, the manifestation of this effect in the temporal and thalamic areas is variable in these previously published studies. For the temporal area, it has been reported that an increase in auditory cortex gray matter related to the presence of tinnitus^37,39^, whereas tinnitus distress related to a decrease in auditory cortex gray matter^36^. Similarly, for the thalamus both increases and decreases in volume were reported^36,38^. Overall, the reported changes in cortical structure that are associated with tinnitus vary between studies, and are in some instances contradictory. The heterogeneity of the tinnitus population, the confound of hearing loss, differences in age as well as non-corresponding techniques and lenient statistical thresholds have been named as contributing factors to this discrepancy^32,40^.

Since emerging evidence indicates that acquired hearing loss and tinnitus are associated with age-related cognitive impairment, the identification of differences in brain structure that are specific to each of these conditions may help us to develop appropriate treatments that not only aid hearing or improve tinnitus, but may additionally prevent cognitive decline. A current challenge is to determine if the structural characteristics that might contribute to cognitive impairment should be attributed to hearing loss, to tinnitus, or related factors such as age. Hence, the aim of this study was to disentangle the effects of age, acquired hearing loss, and tinnitus on the gray matter of the brain.

## Results

**Figure 1:**
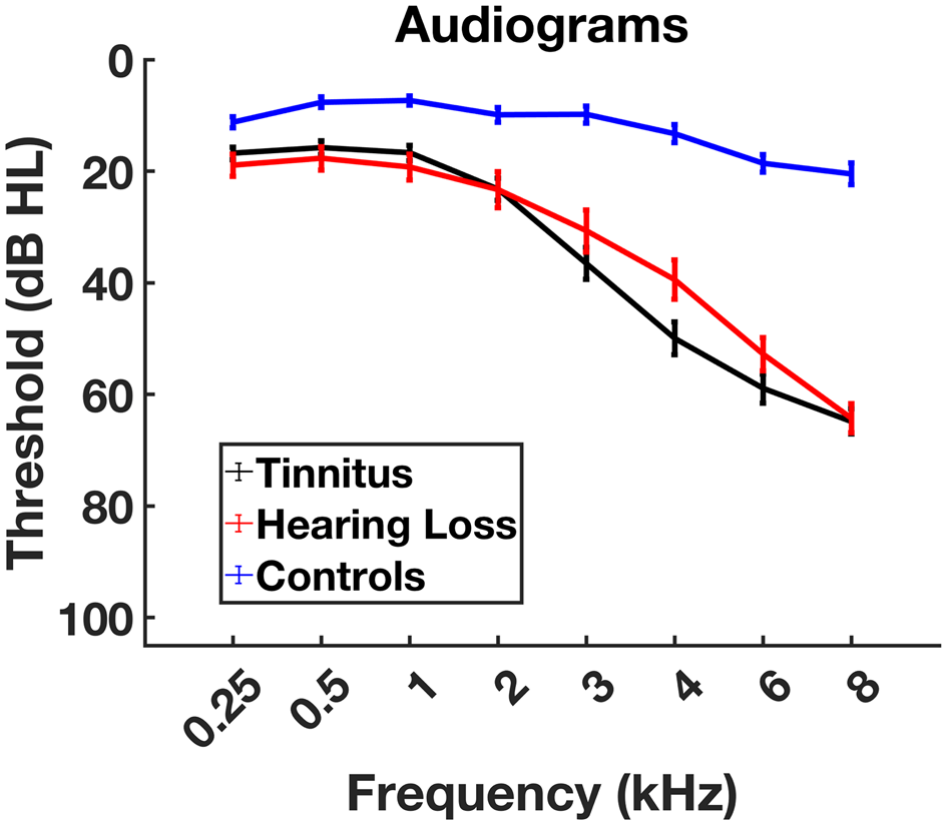
Average hearing thresholds per group with their corresponding standard errors. Pure tone audiometry was used to test octave frequencies from 0.25 to 8 kHz, and 3 and 6 kHz. The two groups with hearing loss, with and without tinnitus, were not significantly different in terms of age (p = .419, t 1.359) and hearing loss [p = .209 (0.25 kHz); p = .3 (0.5 kHz); p = .416 1 (kHz); p = .683 (2 kHz); p = 0.812 (3 kHz); 4 kHz p = .976 (4 kHz); p = .783 (6 kHz); p = .974 (8 kHz)].

**Figure 2:**
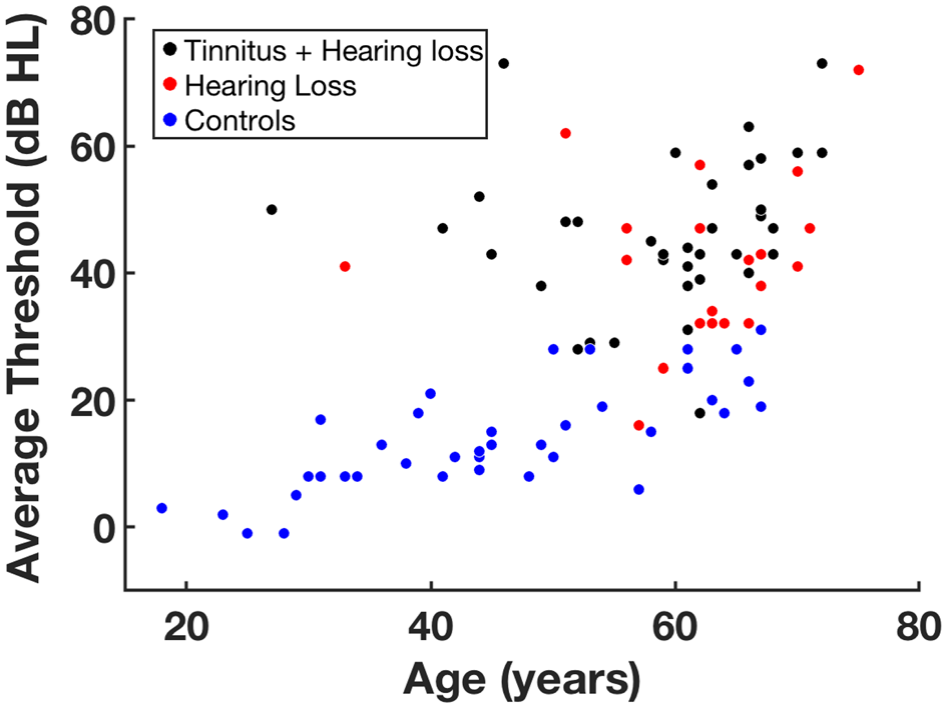
Average hearing threshold of all participants, plotted against age. The control group was significantly younger than both the hearing loss group with (p < 0.0001, t 5.1) and without tinnitus (p < 0.0001, t 5.9). The average threshold was computed for the frequencies of 2, 4, and 8 kHz.

The data of 89 participants are presented, of which 39 had tinnitus and hearing loss (THL group), 21 participants had hearing loss but no tinnitus (HL group), and 39 controls with neither tinnitus and no or minimal hearing loss (CO group). Table 1 contains the demographics of the three participant groups. The mean age of both hearing loss groups differed significantly from the control group whereas the hearing loss groups did not differ from each other. The hearing loss groups, with and without tinnitus, do not differ significantly on any of the frequencies (corrected for multiple comparisons; see description Fig. 1). However, the hearing thresholds of both hearing loss groups differed significantly from those of the control group on all frequencies. Age and average hearing loss (HF-PTA) showed a significant positive correlation across all participants (r = 0.6125, p < 0.0001; Fig. 2). Analysis run on group level showed that only in the control group there was a significant correlation between average hearing loss and age (THL: R = 0.20, p = 0.22; HL: R = 0.10, p = 0.66; CO: R = 0.76, p < 0.0001).

**Table 1:** Demographics of participants. The hearing loss groups, with and without tinnitus, did not differ with respect to age (p = 0.419). The control group was significantly younger than both hearing loss groups, with and without tinnitus (p < 0.0001).

Group	Mean age (years)	Standard deviation	N
Control	45.7	13.5	39
Hearing loss	62.6	8.8	21
Tinnitus + hearing loss	59.2	9.3	39

### Morphometry

#### Voxel based morphometry

**Figure 3:**
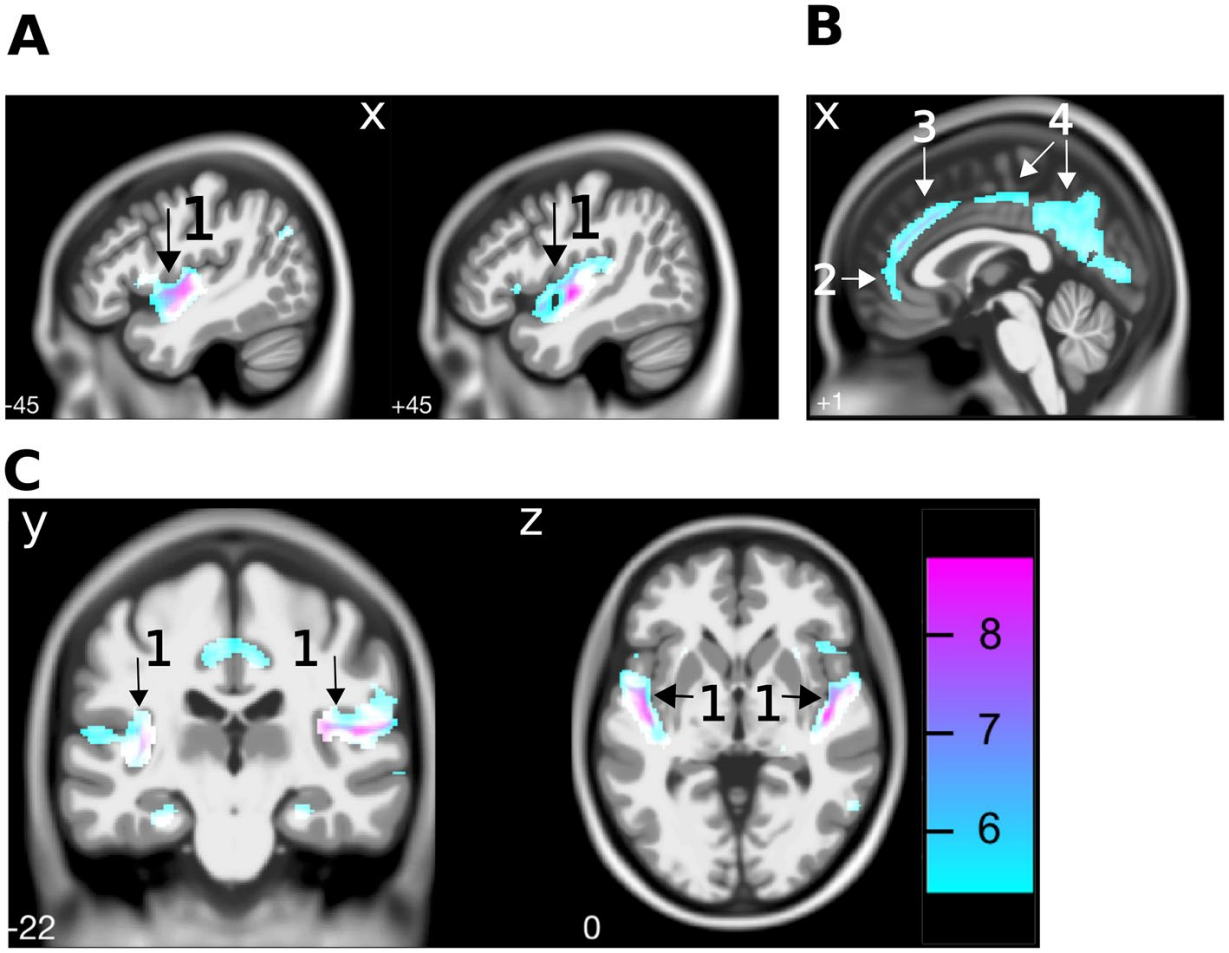
Cortical gray matter volume differences related to age. A multiple regression model with the covariates age, sex, tinnitus, and hearing thresholds at 2, 4, and 8 kHz indicated that increasing age was associated with less gray matter volume in **(A)** temporal areas (including the bilateral auditory cortices (1)) and **(B)** anterior (2), middle (3) and posterior (4) cingulate areas, at FWE < 0.05. Scale bar indicates T values.

A voxel-based multiple regression model was used to explain differences in gray matter volume in terms of age, sex, the presence of tinnitus, and average hearing loss (HF-PTA). In addition, Total Intracranial Volume (TIV) was included as a covariate. This analysis demonstrated that age was a significant predictor for gray matter volume in our study sample (family-wise error (FWE) < 0.05, cluster size k > 20; see Table 2). Specifically, age was inversely related to gray matter volume in the cingulate and auditory cortical areas (see Fig. 3). None of the other predictors were significantly associated with gray matter volume. However, non-significant associations were identified (p < 0.001 uncorrected, k > 20); The presence of tinnitus was associated with greater gray matter volume in the bilateral lingual gyrus, bilateral crus of the cerebellum, left secondary visual cortex, and the left retrosubicular area. High-frequency hearing loss was associated with less gray matter volume in the right cortical auditory areas and the left cerebellar area. Even though these findings were non-significant, they are mentioned here since other studies identified similar areas as related to hearing loss and tinnitus (see Discussion).

**Table 2:** The coordinates for the local maxima of areas that show an inverse relation between the gray matter volume and age.

Area	Cluster		MNI coordinates (mm)		
	P (FWE-cor)	k	X	Y	Z
**Superior temporal gyrus (R)**	< 0.0005	4316	45	− 10	− 3
Insula			48	6	− 8
*Cluster extends to include BA 22 & 41			56	− 2	0
**Superior temporal gyrus (L)**	< 0.0005	3125	− 45	− 6	− 10
Insula			− 46	− 10	2
*Cluster extends to include the insula, BA 22 & 41			− 33	− 21	12
**Putamen (L)**	< 0.0005	382	− 26	9	− 12
*Cluster extends to include the subcallosal gyrus and amygdala			− 26	18	0
**Fusiform gyrus (R)**	< 0.0005	228	24	− 40	− 16
*Cluster extends to include the parahippocampal gyrus fusiform gyrus and lingual gyrus					
**Fusiform gyrus (L)**	< 0.0005	428	− 22	− 44	− 14
Parahippocampal gyrus			− 26	− 28	− 18
Lingual gyrus			− 18	− 46	− 6
**Cingulate gyrus (L)**	< 0.0005	1264	− 14	− 44	42
			9	− 45	33
			− 10	− 51	38
**Middle temporal gyrus (R)**	< 0.0005	123	58	− 57	− 3
**Hippocampus (R)**	< 0.0005	144	22	− 32	− 4
**Calcarine sulcus V1 (R)**	< 0.0005	158	2	− 80	6
*Cluster extends to include the lingual gyrus					
**Mamillary bodies**	< 0.0005	53	0	− 9	− 10
**Middle cingulate gyrus (R)**	< 0.0005	402	3	27	33
Anterior cingulate			2	39	21
			4	16	40
**Posterior cingulate gyrus (R)**	< 0.0005	222	3	− 63	18
**Supramarginal gyrus (R)**	0.001	34	40	− 46	56
**Angular gyrus (L)**	< 0.0005	116	− 46	− 68	32
**Olfactory area (R)**	< 0.0005	104	24	12	− 14
**Putamen (R)**	0.001	31	28	18	2
**Middle temporal gyrus (L)**	0.001	38	− 63	− 46	8
**Inferior frontal gyrus (R)**	< 0.0005	108	44	20	0
BA 45 (R)			54	18	3
*Cluster extends to include the insula and orbitofrontal cortex					
**Superior temporal gyrus (L)**	0.001	44	− 56	− 60	21
**BA 7 (R)**	< 0.0005	67	3	− 64	45
			6	− 63	36

#### Pair-wise comparisons between groups

Pair-wise comparisons on volumetric data of the three participant groups were investigated with Threshold Free Cluster Enhanced (TFCE) two-sample t-tests, that were performed on the modulated gray-matter volume. The pairwise group comparisons tested were: tinnitus and hearing loss versus hearing loss (THL vs. HL), hearing loss versus controls (HL vs. CO), and tinnitus and hearing loss versus controls (THL vs. CO). In addition, the inverse of all these comparisons was tested, resulting in a total of six pair-wise group comparisons. All of the pair-wise comparisons were controlled for age and TIV.

**Figure 4:**
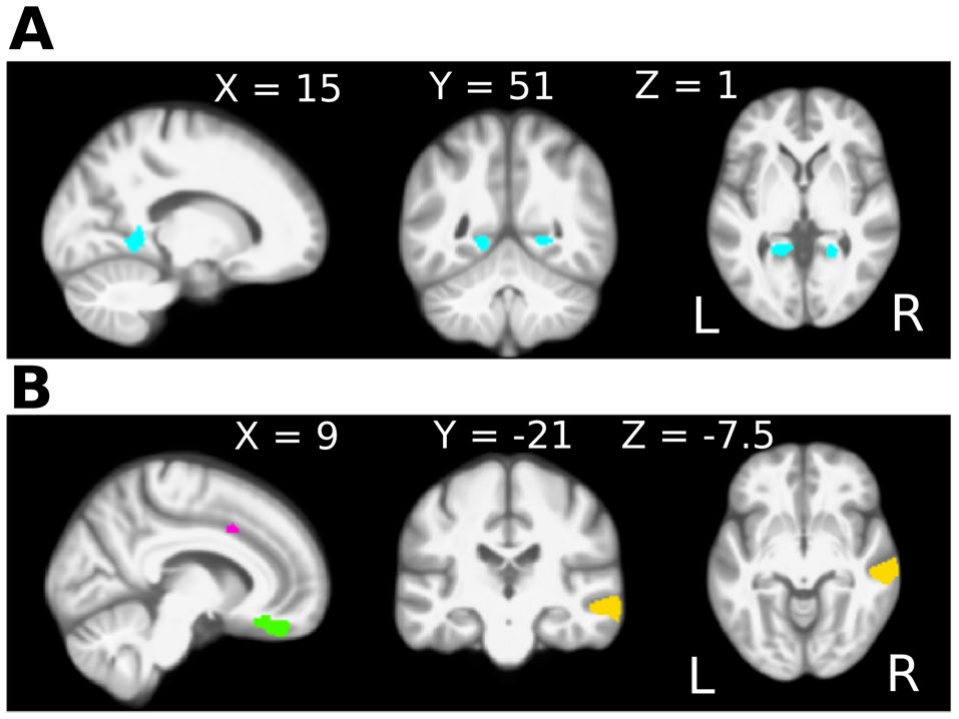
Gray matter volume differences associated with hearing loss and with tinnitus. Threshold free cluster enhancement (TFCE) was employed to investigate group differences in modulated gray matter images. **(A)** Comparison between the hearing loss groups, with and without tinnitus. In the group with tinnitus, larger gray matter volume of the bilateral lingual gyri was observed. These areas are depicted in blue on a medial view of the sagittal plane of the right hemisphere, and a coronal and axial cross section of the entire brain. **(B)** Comparison between the hearing loss group without tinnitus and the control group. In the hearing loss group without tinnitus less gray matter volume was observed in the cingulate cortex (pink), the orbitofrontal cortex (green), and the middle temporal gyri (yellow), depicted on a medial view of the sagittal plane of the right hemisphere. Additional clusters with less gray matter volume, not shown here, were observed in the superior and inferior temporal gyri and the fusiform gyrus (see Table 4). The comparison between the hearing loss group with tinnitus and the controls revealed no significant differences and is therefore not shown in this figure.

The first analysis revealed a difference in gray matter volume between the two hearing loss groups, with and without tinnitus (THL vs. HL). This group comparison showed significantly higher gray matter volume in the bilateral Lingual Gyrus (BA 37, FWE < 0.05, k = 363(L)/277(R)) for the tinnitus group; see Fig. 4A and Table 3. In this comparison, there were no areas that showed significantly smaller gray matter volume. Second, in the hearing loss group without tinnitus, there were no areas in which the volume was larger than in the control group (HL vs. CO). However, gray matter volume was smaller in the bilateral middle temporal gyri, left inferior temporal gyrus, right orbitofrontal area, right cingulate cortex, left fusiform gyrus, and right temporopolar area; see Fig. 4B and Table 4. Finally, in the comparison of hearing loss with tinnitus (THL) versus controls (CO), none of the differences reached significance. See Tables 2, 3, and 4 for the coordinates and cluster sizes of the reported VBM differences.

**Table 3:** The coordinates for the local maxima of gray matter volume differences between the hearing loss groups with and without tinnitus.

Area	Cluster	Peak-cluster	MNI coordinates (mm)		
	P (FWE-cor)	K	X	Y	Z
Lingual gyrus (L)	0.019	363	− 20	− 46	− 3
Lingual gyrus (R)	0.031	277	24	− 46	− 2
	0.039		18	− 44	12
*Cluster extends to include the precuneus					

These areas shows more gray matter volume in the hearing loss group with tinnitus, compared to the hearing loss group without tinnitus. The comparison between the tinnitus group and the controls did not reveal a significant group difference, and is therefore not shown in this table.

**Table 4:** The coordinates for the local maxima obtained via a two-sample t-test, comparing the hearing loss group without tinnitus to the control group.

Area	Cluster	Peak-cluster	MNI Coordinates (mm)		
	P (FWE-cor)	k	X	Y	Z
**Middle temporal gyrus (L)**	0.016	340	− 62	− 33	− 16
*Cluster extends to include the inferior temporal gyrus					
**Middle temporal gyrus (R)**	0.019	1144	63	− 26	− 4
	0.019		57	− 26	− 10
*Cluster extends to include the insula	0.020		66	− 15	− 12
**Sub-gyral area (R)**	0.022	436	10	39	− 21
	0.025		9	30	− 16
*Cluster extends to include the orbitofrontal cortex and amygdala					
**Middle cingulate gyrus (R)**	0.044	44	10	12	39
**Fusiform gyrus (L)**	0.045	59	− 40	− 48	− 22
	0.049		− 34	− 40	− 18
**Superior temporal gyrus (R)**	0.046	70	52	2	− 3
*Cluster extends to include the temporal pole					
**Fusiform gyrus (L)**	0.048	24	− 52	− 46	− 21

The areas identified show less gray matter volume in the hearing loss group without tinnitus compared to the controls.

#### Surface based morphometry

**Figure 5:**
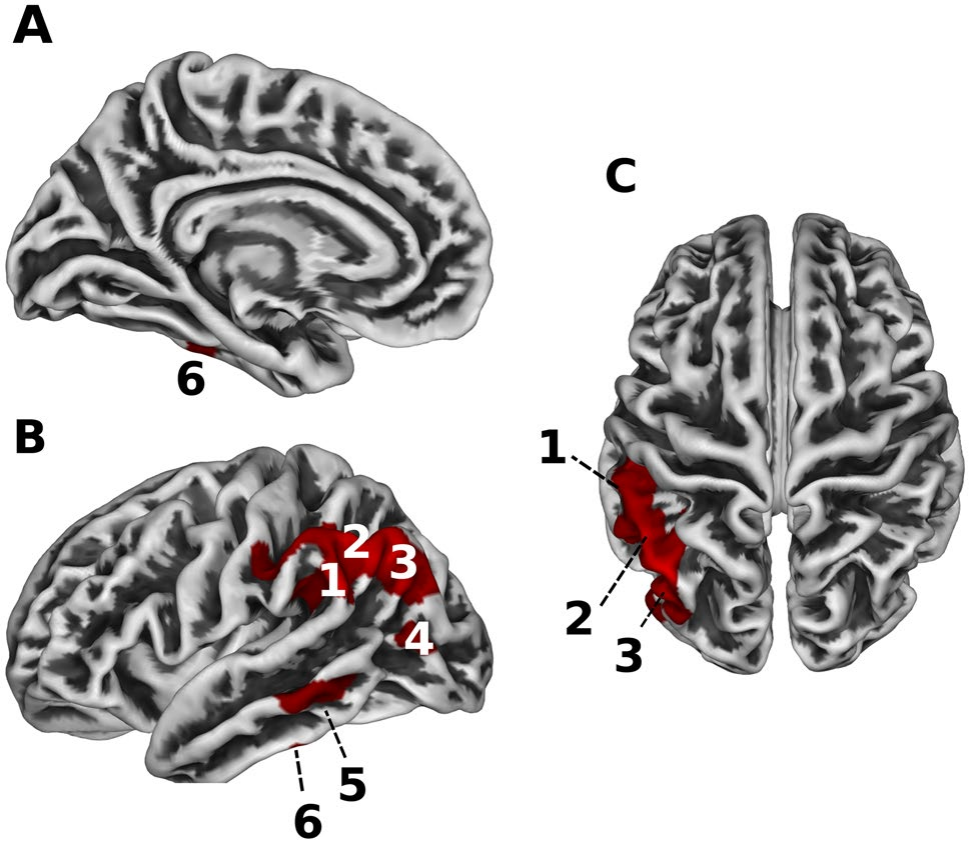
Reduction in cortical surface thickness in the hearing loss group without tinnitus compared to the control group. The three panels show a medial **(A)** and lateral **(B)** view of the left hemisphere, and a top view **(C)** of the entire brain. Areas that contained less cortical surface thickness in the hearing loss group without tinnitus compared to the control group (FWE < 0.05, TFCE) are shown in red. These areas include the supramarginal gyrus (1), the angular gyrus (2), the inferior parietal lobule (3), lateral occipital area (4), and the middle temporal (5) and inferior temporal areas (6), all on the left side of the brain.

In addition to the volumetric measures of the VBM method, surface-based morphometry (SBM) was performed to measure cortical thickness and gyrification^41^. The cortical thickness was significantly reduced in the hearing loss group without tinnitus compared to the control group. This reduction was observed in the left supramarginal gyrus and angular gyrus, extending posteriorly to the inferior parietal gyrus, and the left lateral occipital, middle temporal, and inferior temporal areas (TFCE, FWE < 0.05; see Fig. 5). No significant differences in cortical thickness were observed between both hearing-impaired groups, with and without tinnitus, and between the hearing loss group with tinnitus and the control group. The gyrification analyses did not show any significant differences between the groups.

#### Post-hoc analysis

To assess if the auditory cortex is distinctively affected by age in comparison to other primary sensory or motor related areas, post-hoc region-of-interest (ROI) analyses were performed on both the gray matter volume and the surface-based thickness data, inspired by Profant et al.^29^. Three ROI areas were included: the visual cortex (V1), motor cortex (M1), and the auditory cortex (A1). ROI determination was performed using the HCP_MMP1 atlas for the thickness parcellations, and the atlas provided by Neuromorphometric Inc. for the volume parcellations^42^.

**Figure 6:**
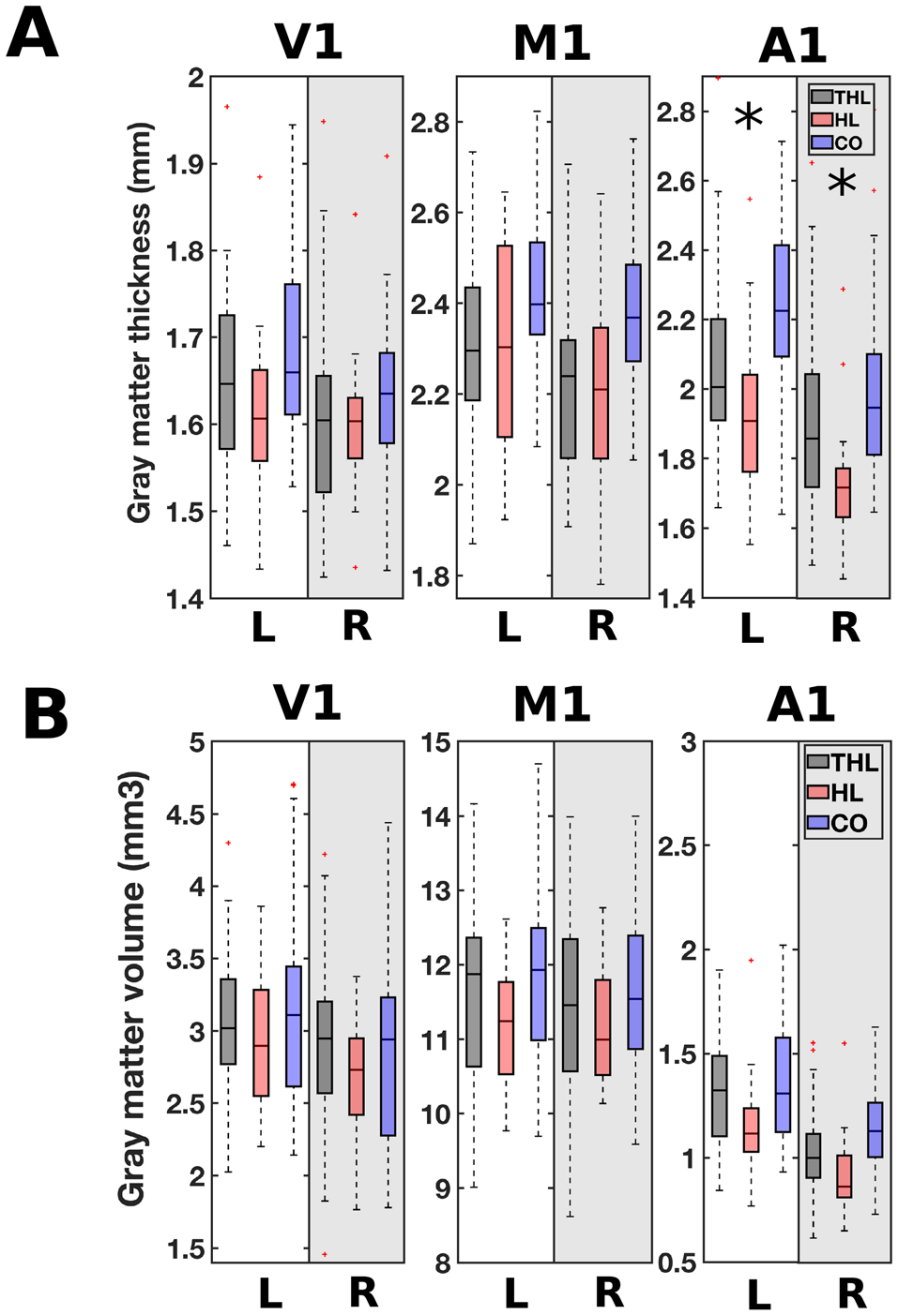
Cortical surface thickness **(A)** and gray matter volume **(B)** in primary visual cortex (V1), primary motor cortex (M1) and primary auditory cortex (A1) for  the three participant groups: THL (hearing loss with tinnitus); HL (hearing loss without tinnitus); CO (controls without tinnitus or hearing loss). The boxplots show the cortical surface thickness and the gray matter volume for both the left (L) and right (R) hemispheres. Significance, corrected for multiple comparisons at a stringent Bonferroni level, is indicated with an asterisk.

The group differences in surface thickness and volume of gray matter that were identified in the three ROIs are depicted in Fig. 6. Left dominant laterality was observed for the auditory cortex, both for the volumetric and surface-based methods. No group related differences in laterality were observed. Therefore, subsequent analyses were performed on the average of left and right ROIs. First, a multivariate analysis of covariance showed that there was a significant decline in both gray matter volume and surface thickness with age in all three ROIs, see Table 5. Second, the effect of group on gray matter volume was tested for the three ROIs included, adjusted for multiple comparisons in the strictest sense via a Bonferroni correction and corrected for age. This analysis showed that there was no significant effect of group on cortical volume or thickness for V1 and M1 after correcting for age (Table 5, Group). Moreover, A1 gray matter volume was not significantly different between the groups. Nonetheless, both the median cortical thickness and the median gray matter volume of A1 were smallest in the hearing-impaired group without tinnitus, and largest in the controls. However, only the thickness measures were significantly different between the groups (see Table 5 and Fig. 6A, right panel). To conclude, the effect of age on all of the included cortical areas is strong, and the only group related difference was observed in the thickness of the auditory cortex.

**Table 5:** The effect of age and group on gray matter volume and thickness in the visual (V1), motor (M1), and auditory cortices (A1).

	Volume		Thickness	
	p-value	F	p-value	F
**Age**				
V1	**0.001**	**12.633**	**0.001**	**12.433**
M1	**0.031**	**4.767**	** < 0.0001**	**19.279**
A1	** < 0.0001**	**32.247**	** < 0.0001**	**17.095**
**Group**				
V1	0.602	0.511	0.775	0.256
M1	0.767	0.767	0.658	0.420
A1	0.112	2.241	**0.048**	**3.137**

Significant results of multivariate analysis of variance are shown in bold. Adjustment for multiple comparisons: Bonferroni. Top: the effect of age on gray matter volume and surface thickness. Bottom: the effect of group on the gray matter volume and surface thickness, with age included as a covariate.

## Discussion

The aim of this study was to disentangle the effect of age, hearing loss, and tinnitus on the structure of cortical gray matter. Three groups were included: (1) hearing loss and tinnitus, (2) hearing loss without tinnitus, and (3) controls without hearing loss or tinnitus. The first result is that age was significantly related to gray matter volume decline in all three groups. Second, in the hearing loss group with tinnitus significantly higher gray matter volume was observed in the lingual gyri compared to the hearing loss group without tinnitus. Third, in hearing loss without tinnitus significant gray matter reductions, relative to controls, were observed in the temporal gyri, orbitofrontal area, cingulate cortex, fusiform gyrus, and the temporopolar area. Additionally, the thickness of the cortical surface was significantly less in the supramarginal gyrus, inferior parietal area, lateral occipital area, middle temporal and inferior temporal areas in hearing loss without tinnitus, compared to controls. These reductions were not observed in hearing loss participants with tinnitus, who were of similar age and had similar hearing thresholds.

### Age related differences

The multivariate analysis of the association between age, hearing loss, and tinnitus showed that age is significantly related to gray matter volume reductions. This effect was observed in the auditory cortices, in the cingulate areas, and in the orbitofrontal area. These specific brain areas have been described in other studies on aging and brain volume^29,43–45^. Our post-hoc analysis on gray matter volume of the visual, motor, and auditory cortices showed a larger effect of age on the auditory than on the visual and sensorimotor cortex. Note that a part of the participants in our study were hearing impaired, either with or without tinnitus. Overall, age appears to have a more detrimental impact on the auditory cortex than it does on the visual or motor cortices. Moreover, hearing loss appears to selectively aggravate the detrimental effect of age on the gray matter of the auditory cortex.

### Hearing loss related differences

In addition to the effect of age, acquired hearing loss has a distinct effect on the gray matter brain volume of the temporal gyri, the orbitofrontal cortex, middle cingulate and fusiform gyrus. Moreover, in the present study hearing loss was associated with differences in the cortical surface thickness of the supramarginal gyrus, angular gyrus, inferior parietal area, occipital and temporal areas. Our results are in agreement with previous reports on reduced gray matter volume in the temporal lobes and cingulate area of individuals with hearing loss^23–26,28^. Similarly, our surface based thickness measure indicated that hearing loss without tinnitus is related to less gray matter thickness in the parietal cortex and insula, which is in line with the reported results of an earlier study^30^. Hearing loss, in addition to age, is thus related to a reduction in the cortical volume and thickness of areas within and beyond the auditory system.

### Tinnitus related differences

Our results support the finding of the study by Husain et al.^24^ that structural changes are more pronounced in hearing loss without tinnitus than in hearing loss with tinnitus. In line with this, the hearing loss group without tinnitus in our study had significantly lower gray matter volume and surface thickness in several areas compared to controls, whereas the hearing loss group with tinnitus did not. Likewise, Husain et al.^24^ reported no significant differences in gray matter volume between hearing loss with tinnitus and healthy controls in participants with hearing thresholds similar to our study. Interestingly, this finding suggests that in hearing loss the additional presence of tinnitus is related to a more limited decline in gray matter and thus better preservation of gray matter in several temporal, frontal, and occipital areas.

In the hearing loss group with tinnitus, the gray matter volume of the lingual gyri was larger than in those without tinnitus. Previously, the lingual gyrus has been connected to tinnitus in studies that investigated resting-state connectivity. These studies reported tinnitus related increases in connectivity of the left lingual gyrus with the left auditory cortex^46^ and decreased connectivity of the left lingual gyrus with the auditory resting state network^47^ compared to controls with normal hearing. Studies that compared tinnitus participants to hearing loss matched controls reported increased connectivity of the bilateral lingual gyrus with the attention and short-term memory networks^48^, increased connectivity of the lingual gyri with the default mode network, and a trend towards decreased connectivity with the auditory resting state network^47^. Our results indicate that in the presence of tinnitus the gray matter volume of the lingual gyrus is altered along with the functional connectivity differences reported in literature. To our knowledge there is currently no research published on specific structural preservation of the lingual gyrus in relation to tinnitus. The precise role of the lingual gyrus in tinnitus remains unclear. Other studies reported that the lingual gyrus is linked to better preserved cognitive function in patients with major depressive disorder^49^, and that larger lingual gyrus volume correlated with better performance on memory tasks^50,51^. In summary, these results suggest a relation between the possible preservation of cognitive functions mediated by the lingual gyrus and the presence of tinnitus.

### Differentiation with other studies on tinnitus

The present study showed that larger gray matter volume in the lingual gyri was specifically related to the presence of tinnitus in individuals with hearing loss. In contrast, previous studies reported tinnitus related reductions in gray matter volume or surface thickness in multiple areas. For instance, differences in the prefrontal cortex^32–37,39^, the supra marginal gyrus^33^, insular cortex^36^, and cingulate area^36^ were reported. Yet, our study showed that changes in these areas were related to age, and differences in the gray matter of the cingulate area were related to hearing loss. These findings are consistent with the results of Wong et al.^27^. In their study it was reported that a significant relation was present between age, not hearing loss, and surface thickness of the superior frontal gyrus. In addition to the aforementioned areas, other studies reported differences in the subcallosal area^35,38^, and the pre and postcentral gyrus^36^ in relation to tinnitus. However, in our study no significant differences were identified that related to these areas in any of our contrasts. In line with previous reports, the right cluster identified in the lingual gyrus expanded to include the precuneus^32^. To elaborate, if age was omitted as a covariate in our analyses or if we used an uncorrected threshold this resulted in the detection of significant volumetric differences related to hearing loss in the aforementioned areas. Since tinnitus patients often have additional hearing loss and are generally older than control groups, these confounds may explain the uncertainty and contradictory reports on structural differences in tinnitus^32,40,52^. Therefore, some of the results described in earlier studies may reflect (small) differences in hearing loss or age, and are not specifically related to the presence of tinnitus.

### Beyond the auditory system: cognition

Our results showed that, regardless of age, several brain areas contained less gray matter in participants with hearing loss, and this effect is more pronounced in hearing loss without tinnitus than in hearing loss with tinnitus. The areas identified in our analyses are in line with areas that are reported to show an aggravated decline of cortical volume^25,26,28^ and surface thickness^30^ in hearing loss and cognitive impairment. Atrophy of several brain areas beyond the primary auditory system has been related to hearing loss, and this widespread loss of cortical gray matter has the potential to affect a wide range of cortical processes that extend beyond auditory processing. The gray matter areas identified in our study are consistent with those reported in previous longitudinal studies that indicated that in age-related hearing loss the decline in these gray matter areas is progressive over time and relates to cognitive impairments^10,28^.

The nature of the relation between hearing loss and cognitive decline is still debated. It has been suggested that a similar mechanism of progressive loss of brain efficiency can result in both central hearing loss and cognitive decline^4,14,53,54^. Directionality of this effect has been suggested, for example the proposal that a decline in central functioning may contribute to poorer speech understanding and thus that cognition can have a big impact on hearing related processes^55^. Moreover, presumably the declining processing speed is the common cause of both cognitive decline and hearing loss, since the auditory system relies on precise timing and a reduction in temporal fine structure can hinder speech processing. On the other hand it has been described that central hearing disorders, or central auditory dysfunctions, often precede cognitive impairment^56^. The latter finding suggests that central hearing dysfunction can be an early marker of cognitive decline. Based on our results, uncompensated hearing loss without tinnitus is linked to a loss of gray matter in several cortical areas that are implicated in cognitive decline, whereas in the presence of tinnitus these areas are better preserved.

Overall, it is important to note that the differences between participant groups may have developed as a result of hearing loss or tinnitus. Alternatively, differences may have existed before tinnitus or hearing loss developed. For example, it is conceivable that some pre-existing morphological characteristics correspond to a susceptibility to develop hearing loss or tinnitus. Based on our study, we cannot distinguish between gray matter differences that developed over time and pre-existing conditions. An additional factor that may influence the outcomes is that none of our participants had hearing aids to compensate for their hearing loss, or tinnitus maskers to alleviate their tinnitus. Hearing aids and tinnitus maskers may impact the brain by generating more input and hence affect cortical plasticity.

### Limitations

The effect of tinnitus on gray matter morphology in this study was derived from the contrast between hearing loss without tinnitus compared to hearing loss with tinnitus. Our results may thus only pertain to tinnitus associated with hearing loss. The possibility exists that tinnitus without the presence of hearing loss affects the gray matter morphology in a different manner.

## Conclusion

This study showed age-related gray matter atrophy in several brain areas, both within and outside of the auditory pathway. With the aid of volumetric and surface-based methods, we showed that age-related hearing loss without tinnitus is related to additional decreases in brain volume of the temporal, frontal, cingular, and parietal areas. The largest differences in gray matter morphometry were caused by age, after which the presence of hearing loss was associated most strongly with a decline in cortical gray matter. Interestingly, the addition of tinnitus in hearing loss individuals appears to reduce the amount of gray matter decline since the gray matter of the hearing loss with tinnitus was not significantly different from that of the control group. The lingual gyrus in particular is larger or better preserved in the presence of tinnitus. These findings confirm that hearing loss has impact beyond the auditory system and is an important factor to consider in the treatment of cognitive impairments.

## Methods

### Participants

One hundred and thirteen participants were included in a larger MRI study at the University Medical Center Groningen, the Netherlands. Three people did not complete all aspects of the research, one data file was corrupted in the transfer process, and in an additional nine scans either the contrast between white and grey matter was insufficient for the segmentation algorithm to work reliably, or movement during anatomical scanning rendered the data with artefacts. This resulted in complete and sufficient data for a total of 99 participants, of which 39 had tinnitus and hearing loss, 21 participants with hearing loss but without tinnitus, and 39 healthy controls with no tinnitus and no or minimal hearing loss. The study was approved by the Medical Ethical Committee of the University of Groningen, performed in accordance with all relevant regulations and informed consent was obtained from all participants.

### Auditory thresholds

The hearing levels of all participants were assessed with pure tone audiometry. Thresholds were determined in a sound attenuating booth for octave frequencies ranging from 0.25 to 8 kHz, and additionally at 3 and 6 kHz. The average hearing loss was quantified as the mean of the hearing thresholds at 2, 4, and 8 kHz of both ears (high-frequency pure-tone average; HF-PTA). A matching procedure was used to estimate tinnitus pitch and loudness. The majority of the participants perceived high frequency tinnitus (n = 35, > 2 kHz), and the remaining four participants reported the perception of broadband noise. None of the participants compensated their hearing loss with hearing aids, or ameliorated their tinnitus with maskers. Participants were required to have hearing thresholds better than 40 dB SPL at 1 kHz to meet the inclusion criteria for a functional task-based scan that was obtained in the same sequence as the anatomical images. This resulted in the inclusion of participants with high frequency hearing loss only.

### MRI data acquisition

MRI scanning was performed at the Neuro Imaging Center in Groningen, on a 3.0 T Philips Intera MRI scanner (Best, the Netherlands). A SENSE 32-channel head coil was used to obtain a whole brain T1 weighted anatomical image with a voxel size of 1 × 1 × 1 mm (TR 10.4 ms, TE 5.7 ms, Acquisition matrix 256 × 200).

### Data analysis

All T1-images were aligned according to the individual anatomical anterior–posterior commissures in Vistasoft-Master, software run in Matlab2018a. Further analyses were done in CAT12, a structural imaging-oriented SPM toolbox^41^, and included segmentation, normalization and smoothing. Total Intracranial Volume (TIV) was estimated for all participants to correct for differences in head and brain size. After these steps were performed, a sample quality check was run on the segmented images to determine if image quality was sufficiently high; all included images scored above 86%. This score is based on a measure of homogeneity of the sample and is used to determine image quality after pre-processing (i.e. mean correlation measure) and it was run with TIV as a nuisance variable. Data with a low mean correlation (i.e. low image quality) or large differences to the mean score were manually checked for sufficient image quality. If this confirmed the low image quality, images were excluded from further analysis.

The included images were smoothed with an 8-mm Gaussian kernel. The subsequent analyses were performed on the modulated gray matter volume images (VBM) and the surface-based thickness measures (SBM). Modulated gray matter images were scaled by the amount of contraction induced by non-linear spatial normalization. The value of each voxel was multiplied with the Jacobian determinant obtained with spatial normalization. After the pre-processing and first level analysis, second level whole group and group wise analyses were performed (Mechelli et al., 2005).

A multiple regression model was run on the modulated gray matter images with sex, age, the presence of tinnitus, and average hearing loss as covariates to check the association between these covariates and the gray matter volume data. Then, two sample t-tests were performed on both the gray matter volume and surface-based thickness measures to test group differences in a pairwise manner. Threshold Free Cluster Enhancement (TFCE) was used to assess the cluster-wise differences in gray matter volume between the groups^57^. This non-linear method can detect both diffuse low-amplitude signals and sharp focal signals. In order to test for statistical significance, TFCE computes p-values for each voxel via permutation testing.

## Data Availability

The dataset analysed in this study is no publicly available due to privacy restrictions. The data can be made available upon reasonable request to the corresponding author.
