# Author Correction: Gray matter declines with age and hearing loss, but is partially maintained in tinnitus

**DOI:** 10.1038/s41598-021-88182-y

**Published:** 2021-04-13

**Authors:** Elouise A. Koops, Emile de Kleine, Pim van Dijk

**Affiliations:** 1 Department of Otorhinolaryngology/Head and Neck Surgery, University Medical Center Groningen, University of Groningen, P.O. Box 30.001, 9700 RB Groningen, The Netherlands; 2 Graduate School of Medical Sciences (Research School of Behavioural and Cognitive Neurosciences), University of Groningen, Groningen, The Netherlands; 3 Cognitive Neuroscience Center Groningen, University of Groningen, Groningen, The Netherlands

Correction to: *Scientific Reports* 10.1038/s41598-020-78571-0, published online 11 December 2020

This Article contains a typographical error in the first paragraph of the Results section, where

“The data of 89 participants are presented, of which 39 had tinnitus and hearing loss (THL group), 21 participants had hearing loss but no tinnitus (HL group), and 39 controls with neither tinnitus and no or minimal hearing loss (CO group).”

should read:

“The data of 99 participants are presented, of which 39 had tinnitus and hearing loss (THL group), 21 participants had hearing loss but no tinnitus (HL group), and 39 controls with neither tinnitus and no or minimal hearing loss (CO group).”

